# Improving laser standards for three-photon microscopy

**DOI:** 10.1117/1.NPh.8.1.015009

**Published:** 2021-03-06

**Authors:** Deano M. Farinella, Arani Roy, Chao J. Liu, Prakash Kara

**Affiliations:** University of Minnesota, Department of Neuroscience and Center for Magnetic Resonance Research, Minneapolis, Minnesota, United States

**Keywords:** three-photon, microscopy, lasers

## Abstract

**Significance:** Three-photon excitation microscopy has double-to-triple the penetration depth in biological tissue over two-photon imaging and thus has the potential to revolutionize the visualization of biological processes *in vivo*. However, unlike the plug-and-play operation and performance of lasers used in two-photon imaging, three-photon microscopy presents new technological challenges that require a closer look at the fidelity of laser pulses.

**Aim:** We implemented state-of-the-art pulse measurements and developed innovative techniques for examining the performance of lasers used in three-photon microscopy. We then demonstrated how these techniques can be used to provide precise measurements of pulse shape, pulse energy, and pulse-to-pulse intensity variability, all of which ultimately impact imaging.

**Approach:** We built inexpensive tools, e.g., a second harmonic generation frequency-resolved optical gating (SHG-FROG) device and a deep-memory diode imaging (DMDI) apparatus to examine laser pulse fidelity.

**Results:** First, SHG-FROG revealed very large third-order dispersion (TOD). This extent of phase distortion prevents the efficient temporal compression of laser pulses to their theoretical limit. Furthermore, TOD cannot be quantified when using a conventional method of obtaining the laser pulse duration, e.g., when using an autocorrelator. Finally, DMDI showed the effectiveness of detecting pulse-to-pulse intensity fluctuations on timescales relevant to three-photon imaging, which were otherwise not captured using conventional instruments and statistics.

**Conclusions:** The distortion of individual laser pulses caused by TOD poses significant challenges to three-photon imaging by preventing effective compression of laser pulses and decreasing the efficiency of nonlinear excitation. Moreover, an acceptably low pulse-to-pulse amplitude variability should not be assumed. Particularly for low repetition rate laser sources used in three-photon microscopy, pulse-to-pulse variability also degrades image quality. If three-photon imaging is to become mainstream, our diagnostics may be used by laser manufacturers to improve system design and by end-users to validate the performance of their current and future imaging systems.

## 1 Introduction

The now ubiquitous field of two-photon microscopy has been used to visualize a wide array of biological phenomena *in vivo*, from imaging neurons in healthy animals^1^^–^^3^ and in disease models such as Alzheimer’s,^4^ to imaging the movement of fluorescently labeled viruses invading the lungs^5^^,^^6^ and the mechanisms of glomerular filtration in the kidneys.^7^ Three-photon microscopy builds on the success of two-photon microscopy using longer excitation wavelengths to reduce light scattering and a three-photon absorption process to achieve better nonlinear confinement in biological tissue, resulting in higher signal to background ratio at larger depths.^8^^–^^12^ However, three-photon excitation also poses unique challenges due to its heightened sensitivity to the fidelity of the laser source.

Since three-photon excitation requires the near-simultaneous absorption of three photons, laser pulses with higher photon density (higher energy and shorter pulse duration) are used when compared to two-photon microscopy. As a result of using higher energy pulses, lower repetition rates must be used to manage the amount of heat delivered to the sample. Consequently, the integrated fluorescence signal used to construct imaging pixels is a result of fewer excitation pulses. Thus, pulse-to-pulse intensity fluctuations on the timescale of microseconds to milliseconds to seconds will degrade the imaging quality of biological samples. Additionally, because the three-photon absorption rate is proportional to intensity cubed,^13^ distortion of individual pulses in the excitation source can detract from excitation efficiency. Therefore, examining time-dependent biological processes that produce meaningful changes in fluorescence brightness requires a stable, distortion-free three-photon excitation source.

## 2 Methods

### 2.1 Simulating Measurements of Laser Pulses with Various Phase Distortions

**Fig. 1:**
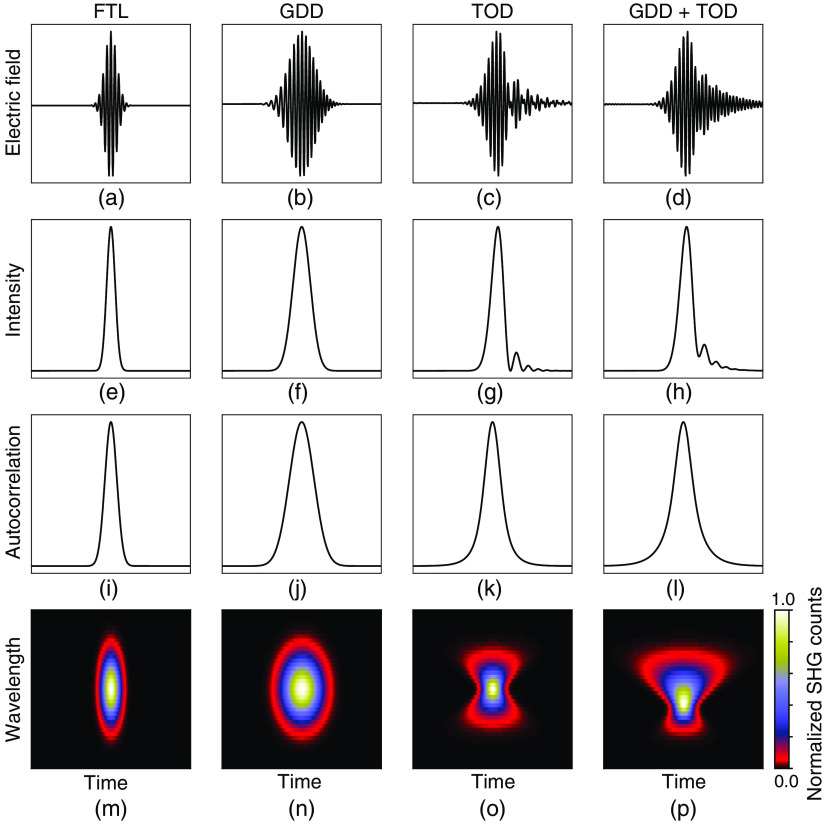
Simulations of distortions of an ideal Gaussian pulse shape caused by GDD and/or TOD. (a)–(d) Electric field. (e)–(h) Laser temporal intensity envelope. (i)–(l) Simulated intensity autocorrelation. (m)–(p) SHG spectrogram. (a), (e), (i), and (m) Laser pulses at the FTL display an ideal pulse shape, where the instantaneous wavelength of the electric field is fixed and the temporal intensity envelope is minimized, leading to a Gaussian shaped intensity autocorrelation and elliptical SHG spectrogram. (b), (j), (f), and (n) Laser pulses with GDD show a change in instantaneous wavelength of the electric field as a function of time, and a broadening of the temporal intensity envelope, leading to a broadened Gaussian intensity autocorrelation and elliptical SHG spectrogram. (c), (g), (k), and (o) Laser pulses with TOD show a beating of the electric field of the laser pulse and a modulated temporal intensity envelope outside of the central peak, resulting in an intensity autocorrelation of a different shape and an SHG spectrogram that takes on an hourglass shape. (d), (h), (l), and (p) Laser pulses with both GDD and TOD exhibit a combination of these effects leading to an intensity autocorrelation with accentuated wings and an hourglass shaped SHG spectrogram with an asymmetric broadening due to the GDD.

Gaussian-shaped laser pulses were analytically defined with no phase distortions, group delay dispersion (GDD), third-order dispersion (TOD), and a combination of the two to show the resulting shape of their electric field and temporal intensity envelope shown in Figs. 1(a)–1(d) and Figs. 1(e)–1(h), respectively. To explore the differences in measuring these laser pulses with different techniques, autocorrelation and second-harmonic generation frequency resolved optical gating (SHG-FROG) measurements were then simulated by calculating the analytical intensity autocorrelation, and computing the SHG spectrogram in Figs. 1(i)–1(l) and Figs. 1(m)–1(p), respectively.

### 2.2 Measuring the Duration of Laser Pulses for Three-Photon Microscopy

Laser pulses from a 1 MHz Spirit-non-collinear optical parametric amplifier (NOPA) (set at 1300 nm central wavelength, Spectra Physics) were characterized by a home-built scanning SHG-FROG device^14^ [see Fig. S1 in Supplementary Material]. The laser pulses were attenuated by uncoated fused silica wedges (PS811, Thorlabs) and further attenuated by an achromatic half-wave plate (AHWP05M-1600, Thorlabs) and SF2 polarizing beam splitter (05FC16PB.9, Newport). The leftover white light in the NOPA laser profile was cleaned with a long-pass filter (FELH1150, Thorlabs) to remove beam profile pollution and simplify alignment. For FROG measurements of the entire width of the laser beam, the beam was down-collimated by a 2× C-coated telescope (GBE02-C, Thorlabs). After down collimation and attenuation, the laser beam was aligned into the entrance irises of the SHG-FROG with gold mirrors (10D20ER.4, Newport).

In the SHG-FROG device, the laser pulses were split into two beam paths (a reference path and a delay path) by a pellicle beam splitter (PBS-2C, Newport). The delay path had a variable path length by varying the position of a retro-reflecting mirror pair on a delay stage. The laser pulses from each path were steered by gold mirrors (10D20ER.4, Newport) onto the surface of a f=50  cm mirror (20DC1000ER.2, Newport), which spatially overlapped the laser pulses in a type I β-barium borate crystal (BBO, United Crystal). The temporal overlap was achieved by varying the length of the delay path to generate SHG. The SHG signal was then directed by a mirror (10D20ER.2, Newport) through a B-coated f=10  cm lens (LA1509-B, Thorlabs) to eventually focus into the slit of a spectrometer (Flame-S-VIS-NIR, Ocean Optics).

FROG traces were taken by spectrally resolving the SHG signal from laser pulses overlapped in time and space inside the BBO crystal. Custom LabVIEW software was used to send commands to the motorized actuator (CONEX-LTA-HS, Newport) to perform the scan and record the spectrum at each delay point, which constructed the FROG trace/spectrogram. We took 201 spectra spaced by ∼1.9  fs to create the spectrogram.

### 2.3 FROG Phase-Retrieval Algorithm

**Fig. 2:**
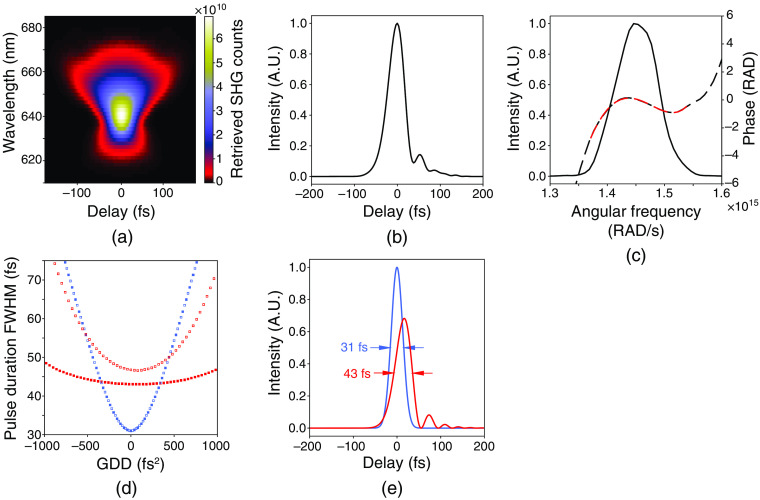
Measurements of spectral intensity and phase by SHG-FROG and modeling of laser pulses. (a) The retrieved SHG spectrogram. (b) The temporal intensity envelope. (c) Spectral intensity and phase. The black solid line represents the laser spectral intensity, the dashed black line represents the spectral phase, and the dashed red line represents a polynomial fit to the measured phase. (d) Analytical modeling of the effects of TOD on pulse compression and measurement by autocorrelation. Data shown as red squares shows the effects of adding or removing GDD from Gaussian laser pulses with the TOD measured from our laser source ($∼22,750\;{\mathrm{fs}}^{3}$). Data shown as blue squares shows the effects of adding or removing GDD from Gaussian laser pulses with no TOD. Filled squares represent the actual pulse duration and unfilled squares are the pulse duration from a simulated autocorrelation measurement. (e) Modeling the effects of TOD on the temporal intensity envelope. A comparison of the temporal intensity envelope of a Gaussian laser pulse with the TOD measured from our laser shown in red and a Gaussian laser pulse with no TOD shown in blue, assuming $\mathrm{GDD}=0\;{\mathrm{fs}}^{2}$.

Data taken with the scanning SHG-FROG was then processed to retrieve the spectral phase.^15^ The retrieval of the spectral phase from the measured SHG spectrogram allows for the determination of the underlying shape of the laser pulse temporal intensity envelope [e.g., Figs. 2(a)–2(c)]. Data were extracted from the measured spectrogram in the 600- to 700-nm wavelength range, centered at the second harmonic, i.e., at half the central wavelength of the laser. Then the data were padded with zeros at 50% of the full width and ultimately binned to a grid of 256× 256  pixels . The phase retrieval algorithm was executed on the binned data until a FROG error of $g=6.9\times 10^{-3}$ was reached.

### 2.4 Determination of Spectral Phase Coefficients from FROG Measurements

The measured spectral phase as a function of frequency [e.g., Fig. 2(c)] can be approximated by a Taylor series expansion with respect to the central frequency of the laser spectrum $\omega_{0}$ to obtain the following Eq. (1).

$$\varphi (\omega )=\varphi_{0}+\frac{\varphi^{\prime }(\omega_{0})}{1!}(\omega -\omega_{0})+\frac{\varphi^{\prime \prime }(\omega_{0})}{2!}{\left(\omega -\omega_{0}\right)}^{2}+\frac{\varphi^{\prime \prime \prime }(\omega_{0})}{3!}{\left(\omega -\omega_{0}\right)}^{3}+\ldots .$$ (1)

Here, ω is the angular frequency, and φ is the spectral phase. The zeroth-order term and first-order term result in a shift in the carrier envelop phase and a group delay respectively, and consequently do not contribute to changes in pulse width. The quadratic term is known as the GDD and is the first term in the expansion that results in pulse broadening. GDD can be compensated with many common pulse compressor designs (i.e., prisms, diffraction gratings, and bulk material) and is defined as, $\mathrm{GDD}\equiv \varphi^{\prime \prime }(\omega_{0})$. Phase coefficients beyond GDD are typically referred to by their order [i.e., $\mathrm{TOD}\equiv \varphi^{\prime \prime \prime }(\omega_{0})$ and fourth-order dispersion $\left(\text{FOD})\equiv \varphi^{\prime \prime \prime \prime }(\omega_{0}\right)$ etc.]. These higher-order terms are generally costly and difficult to compensate in the required amount while simultaneously compensating for GDD, especially for the high repetition lasers used in multi-photon imaging.

To determine the GDD and TOD from the FROG measurement, the measured spectral phase was fit by a fourth-order polynomial centered at the peak wavelength of the retrieved spectrum [e.g., Fig. 2(c)]. The fit extended to where the retrieved spectral intensity fell to ∼5% of the peak intensity and was used to compute the GDD and TOD coefficients as described in Eq. (1). Due to the arrow of time ambiguity in SHG-FROG, the sign of the GDD and TOD was determined empirically by introducing increments of a material with a known GDD and TOD into the path and taking additional SHG-FROG measurements to determine if the GDD and TOD increased or decreased with the introduction of the material. This approach revealed the sign of GDD to be negative, and the sign of TOD to be positive.

### 2.5 Analytical Modeling of the Effects of GDD and TOD on Laser Pulses

**Fig. 3:**
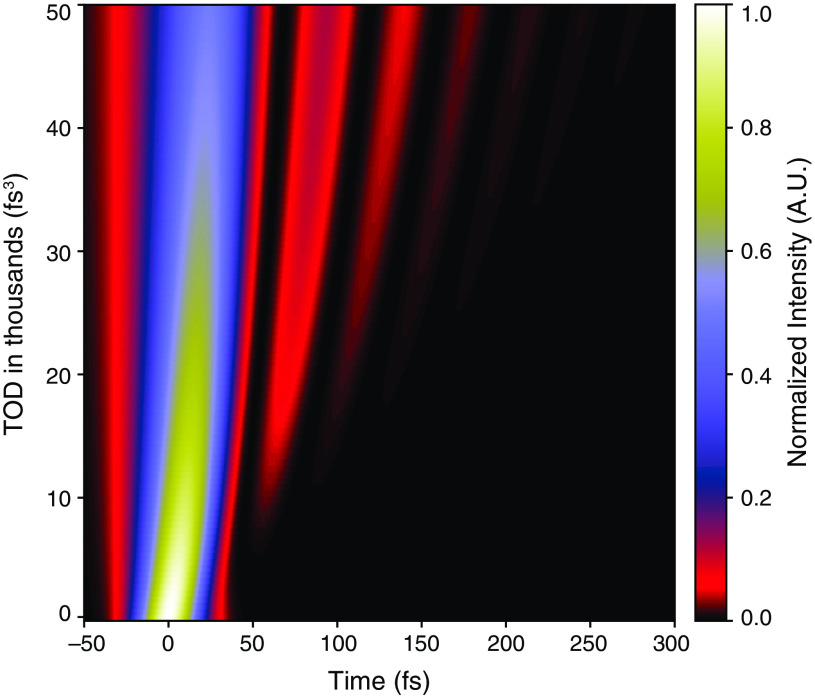
Effects of TOD on temporal energy distribution in a GDD compensated laser pulse. For our model pulse, which was 31 fs FTL, the temporal intensity envelope on the horizontal axis is shown for a range of TOD values along the vertical axis, assuming no other phase distortions, i.e., $\mathrm{GDD}=0\;{\mathrm{fs}}^{2}$. Here, pulse energy is held constant as TOD is increased to visualize its effect on peak intensity at a given pulse energy.

To analytically model the effects of GDD and TOD on the temporal intensity envelope of our laser pulses, we first measured the spectrum of the Spirit-NOPA laser beam using a spectrometer (NIRQUEST, Ocean Optics). This spectrum was used to calculate the analytical inverse Fourier transform (IFT), which determines the Fourier transform limit (FTL) (the shortest pulse that could be supported by the bandwidth) of the laser spectrum. Signals that fell below 1% of the peak signal in the measured spectrum were set to zero and padded with zeros. The full width at half maximum (FWHM) of the resulting IFT was rounded to the nearest femtosecond and determined to be ∼31  fs. Therefore, this is the shortest possible pulse that could be supported by our laser spectrum, representing a pulse with no spectral phase distortion. A Gaussian spectrum (80-nm FWHM) representing a compressed laser pulse with a Gaussian shape and FWHM of 31 fs was used as an analytical model for exploring the effects of GDD and TOD in all analytical simulations [e.g. Figs. 2(d)–2(e), 3 and Fig. S2 in Supplementary Material].

The effects of GDD on model laser pulses with and without the measured TOD was determined using the open-source python package PyNLO, which has the capability of analytically modeling the laser pulses with arbitrary spectral phase.^16^ The effects of TOD on pulse compression was determined by adding the measured TOD from our laser source to the analytical Gaussian model pulses (∼31  fs). Then, to emulate pulse compression, these pulses (with fixed TOD) were given differing amounts of GDD in the range −1000 to $+1000\;{\mathrm{fs}}^{2}$ [e.g., Fig. 2(d)]. The effect of TOD alone on the ideal pulse profile (which implies all GDD has been removed) was explored by adding exactly the measured amount of TOD to the model pulse, and compared to the model pulse itself [e.g., Fig. 2(e)]. Similarly, the effects of scaling TOD on pulse duration were demonstrated by adding a range of TOD values from 0 to $50,000\;{\mathrm{fs}}^{3}$ to our model pulses and recording their FWHM pulse duration. Simulated autocorrelation measurements were performed by analytically autocorrelating the temporal intensity envelope of the model pulses and subsequently deconvolving the autocorrelation by a Gaussian deconvolution factor ($\sqrt{2}$). This simulates an intensity autocorrelation measurement in which the underlying pulse shape is unknown but assumed to be Gaussian. To explore the effects of TOD on energy distribution in a pulse, we also analytically simulated the temporal intensity envelope with a range of TOD values [e.g., Fig. 3].

### 2.6 Determining Expected Brightness from Simulation Results

Three-photon signal photons per pulse (i.e., brightness) was calculated using Eq. (2) below, as originally described by Wang and Xu.^17^

$$3\text{PE photons per pulse}=\frac{1}{3}\frac{g_{p}^{\left(3\right)}}{\tau^{2}}\phi C(\eta \sigma_{3})n_{0}\frac{2\pi^{2}}{3\lambda_{3p}^{3}}{\left(\frac{\lambda_{3p}}{\pi w_{0}}\right)}^{2}{\left(\frac{P_{3p}}{f}\right)}^{3}.$$ (2)

**Fig. 4:**
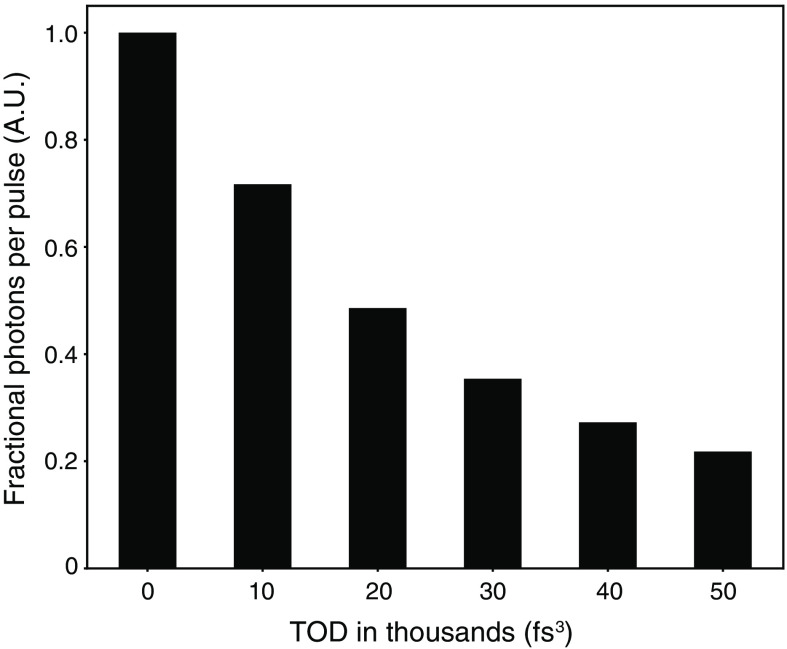
Three-photon signal brightness evaluated at a range of TOD values for GDD compensated pulses. Our model Gaussian laser pulse with ∼31  fs FTL was assigned a range of discrete TOD values with no other phase distortions. The resulting pulse intensity profiles were used to calculate the expected signal photons per pulse, and normalized to the case with no TOD to give fractional photons per pulse. Average power is held constant to show the effect of TOD on image brightness at a fixed average power.

Here, $g_{p}^{\left(3\right)}$ is the third-order coherence factor; τ is the FWHM pulse length; ϕ is the collection efficiency; C is the fluorophore concentration; $\eta \sigma_{3}$ is the action cross-section of the fluorophore; $n_{o}$ is the refractive index of the medium; $\lambda_{3p}$ is the laser wavelength; $w_{0}$ is the beam waist; $P_{3p}$ is the laser average power; and f is the laser repetition rate. Our simulated laser pulses described in the previous section were used to determine the unique third-order temporal coherence factor $g_{p}^{\left(3\right)}$ and FWHM pulse length τ at a range of TOD values holding all other parameters constant. To obtain the fractional photons per laser pulse, the resulting 3PE photons per pulse was normalized to the case of no TOD [e.g., see Fig. 4]. When multiple laser bandwidths were investigated, all values were also normalized to the case of no TOD with the broadest bandwidth [see Fig. S3 in Supplementary Material].

### 2.7 Microscope Imaging

Imaging was performed with a commercial multiphoton microscope (Investigator, Bruker Nano), which we further customized for three-photon imaging. The idler output (tuned to 1300 nm central wavelength) from a NOPA-VISIR (Spectra Physics) pumped by a 1 MHz 70 W laser (Spirit 1030-70, Spectra Physics) was used as the three-photon excitation source. This two-stage optical parametric amplifier is the same design as that used in other commercial laser systems for three-photon imaging such as the Coherent Monaco-OPERA pumped by a different source laser. All imaging of fluorescent slides was performed using a 25× objective lens (XLPLN25XWMP2, Olympus). The dispersion of laser pulses was compensated by a prism compressor in the optical path before the microscope. The emitted photons were transmitted through a short-pass primary dichroic mirror (t770spxrxt, Chroma) and further filtered by a short-pass filter (ET770sp-2p-1500IR, Chroma) before being reflected by long-pass dichroic mirror (T565lpxr, Chroma) and ultimately transmitted through a bandpass filter (525±25  nm bandpass, Chroma) before being collected by a photomultiplier tube detector (H10770PB-40 SEL, Hamamatsu).

### 2.8 Deep-Memory Diode Imaging

Laser pulses from the 1 MHz Spirit-NOPA tuned to 1300 nm central wavelength were attenuated by a half-wave plate (05RP02-40, Newport) and SF2 polarizing beam splitter (05FC16PB.9, Newport) to decrease the pulse energy, as needed. Laser pulses were then focused with a 16× microscope objective (N16XLWD-PF, Nikon) onto the surface of a GaP photodiode (DET25K2 or PDA25K2, Thorlabs) to induce three-photon absorption signals [Fig. S4(a) in Supplementary Material]. For one-photon DMDI, the measurement does not require a focusing objective lens, and in this case an InGaAs photodiode was used (DET05D2, Thorlabs) [see Fig. S4(c) in Supplementary Material].

Laser power was adjusted to achieve similar three-photon signal strengths in each category of the DMDI measurements of stable pulses, microsecond-scale fluctuations, and millisecond-scale variations [see Fig. 6]. An example raw voltage trace from the GaP photodiode trace is included and was achieved at an incident average power of 18.4 mW [Fig. S5 in Supplementary Material].

The photodiode signals were recorded by a high buffer memory oscilloscope (Picoscope 6404 or Picoscope 3406 DMSO, Picotech) at 62.5 megasamples per second or higher. When using the GaP photodiode and a 50  Ω terminating resistor, the rise and fall time of the voltage transient was ∼55  ns. This allowed for seven or more samples per diode transient. The peaks in the diode voltage traces were identified and every three signal peaks were summed and plotted two ways [see Fig. 6]. Summing three successive voltage peaks from the photodiode measurements was only done so as to mimic the 3  μs integration time we use in microscope imaging experiments.

### 2.9 RMS% Laser Stability

The root-mean-square (RMS) is defined as Eq. (3),

$$\mathrm{RMS}=\sqrt{\frac{1}{N}\overset{N}{\underset{i}{\sum }}{\left(x_{i}\right)}^{2}},$$ (3)

where N is the number of measurements and $x_{i}$ is an individual measurement. On the other hand, the RMS% is defined as Eq. (4),

$$\mathrm{RMS}\%=(\frac{1}{\overset{¯}{x}}\sqrt{\frac{1}{N}\overset{N}{\underset{i}{\sum }}{\left(x_{i}-\overset{¯}{x}\right)}^{2}})\times 100,$$ (4)

where $\overset{¯}{x}$ is the mean of all the measurements. RMS% is similar to the coefficient of variation and is readily identified as the ratio of the standard deviation to the mean, expressed as a percentage.

## 3 Results and Discussion

### 3.1 Factors that Limit Obtaining the Shortest Possible Laser Pulse

Due to their large bandwidth, laser sources used for three-photon microscopy are more challenging to harness than those used for two-photon microscopy. The bandwidth of laser pulses determines the shortest ideal pulse that can be achieved, known as the FTL, where larger bandwidths provide the potential for shorter pulses. The FTL is achieved when the peaks of the constituent wavelengths that make up the bandwidth of the laser pulse are coincident. If these constituent wavelengths have a nonzero phase shift with respect to each other, the laser pulse they construct will be distorted from its ideal shape. The phase of the constituent wavelengths (spectral phase) can be mathematically approximated by a polynomial, where the first two terms that affect the redistribution of pulse energy are GDD and TOD [see Sec. 2 and Eq. (1)]. Laser pulses with larger bandwidths are more sensitive to phase distortions than laser pulses with smaller bandwidths. For example, an ideal 100 fs Gaussian laser pulse (typical for two-photon excitation) with $∼1000\;{\mathrm{fs}}^{2}$ of GDD would broaden to ∼104  fs. Conversely, an ideal 30 fs Gaussian laser pulse (typical for three-photon excitation) with $∼1000\;{\mathrm{fs}}^{2}$ of GDD would more than triple to ∼97  fs. Because the three-photon absorption process is more sensitive to changes in peak intensity, the larger susceptibility to broadening of the three-photon pulse will have a more dramatic effect on imaging when compared to laser pulses intended for two-photon excitation. Therefore, in three-photon applications, more attention must be paid to these phase distortions that could dramatically change the pulse properties.

### 3.2 More Accurate Measurement of Laser Pulse Properties for Three-Photon Microscopy

#### 3.2.1 Simulated effects of phase distortions on laser pulses

Based on the above-mentioned theoretical principles that govern pulse duration, we first simulated the pulse distortion effects of GDD and TOD. Laser pulses at the FTL display an ideal pulse shape, where the instantaneous wavelength is fixed and the temporal intensity envelope is minimized [Figs. 1(a) and 1(e)]. Pulses with GDD show a change in the instantaneous wavelength as a function of time and a broadened temporal intensity envelope [Figs. 1(b) and 1(f)]. This phase distortion can be compensated with pulse compressors consisting of prism or grating pairs to approach the FTL. However, pulses with TOD show an asymmetric beating of the electric field and modulated temporal intensity envelope [Figs. 1(c) and 1(g) and Figs. 1(d) and 1(h)]. Such asymmetric distortions are more challenging to correct. Furthermore, TOD can be difficult to detect and is impossible to quantify when using intensity autocorrelations since they cannot uniquely determine the laser electric field or the intensity profile of laser pulses.^18^ For example, the simulated intensity autocorrelations of Gaussian laser pulses with TOD could be incorrectly interpreted as pulses with no underlying phase distortions but a slightly different underlying pulse shape [Figs. 1(i)–1(l)]. Techniques such as SHG-FROG are designed to quantify phase distortions missed by autocorrelation (see Sec. 2). Specifically, the SHG spectrogram can be used to reconstruct the electric field and intensity profile of the underlying laser pulse [Figs. 1(m)–1(p)]. Ideal laser pulses at the FTL and laser pulses with GDD have SHG spectrograms with an elliptical shape, where an increase in temporal width is indicative of pulse broadening due to GDD [Figs. 1(m) and 1(n)]. Most critically, in the presence of TOD, the SHG spectrogram has an hourglass shape, where adding GDD leads to an asymmetric broadening of the hourglass shape [Figs. 1(o) and 1(p)].

#### 3.2.2 Comprehensive measurement of laser pulse properties

To directly measure the phase in our laser pulses, we built an SHG-FROG^14^ [see Sec. 2 and Fig. S1 in Supplementary Material]. The SHG-FROG measurements in Figs. 2(a) and 2(b) show a spectrogram and temporal pulse shape qualitatively consistent with the presence of GDD and TOD as observed in the simulations shown in Figs. 1(h) and 1(p), respectively. To quantify the amount of GDD and TOD, the measured spectral phase can be fit with a polynomial expanded about the central frequency [dashed black line in Fig. 2(c) and see Sec. 2 and Eq. (1)]. The second-order term is used to calculate the GDD, which for our laser system was $∼-590\;{\mathrm{fs}}^{2}$. The third-order term is used to calculate the TOD, which for our laser was $∼22,750\;{\mathrm{fs}}^{3}$. Because the goal was to estimate the amount of TOD in the laser source, we performed these measurements at the laser head [see Fig. S1 in in Supplementary Material] rather than after passing through the table optics, microscope, and objective lens, which would add more TOD.^19^

### 3.3 Consequences of Laser TOD for Three-Photon Imaging

#### 3.3.1 Effect of laser TOD on obtaining the shortest laser pulses

We added and removed GDD from a model Gaussian laser pulse containing TOD in the amounts measured from our laser source and compared these profiles to a model Gaussian pulse with no TOD [Fig. 2(d)]. When all GDD is removed from the laser pulse with TOD, it can only be compressed to ∼43  fs, as opposed to ∼31  fs if there was no TOD. Furthermore, in the presence of TOD, the duration of this pulse from autocorrelations deviates strongly from the actual pulse duration [compare open and filled red data points in Fig. 2(d)]. Whereas, in the absence of TOD, the pulse durations would otherwise match identically [see blue data points in Fig. 2(d)]. Thus, the ability to compress laser pulses by conventional methods and the accuracy of the intensity autocorrelation in determining pulse duration both deteriorate as a function of TOD. In essence, this deterioration is due to the asymmetric redistribution of energy in the laser pulse [see Fig. S2 in Supplementary Material].

#### 3.3.2 Effects of laser TOD on fluorophore brightness and heating of the sample

The leftover asymmetric redistribution of energy due to TOD (described above) will limit the achievable peak intensity and therefore imaging brightness in three-photon imaging. Laser pulses with our measured TOD versus no TOD (both with the same pulse energy) were analytically modeled and the case with TOD shows a decrease in peak intensity by ∼32% when compared to the case with no TOD [Fig. 2(e)]. In the instance of actual three-photon imaging, where laser pulses traverse through the rest of the path (table optics and microscope), the pulses would accumulate even more TOD, which would further limit their peak intensity even after all GDD was removed [see Fig. 3]. This implies that laser pulses with large TOD will need to operate at a higher average power to achieve the same brightness, when compared to laser pulses without TOD.

To quantify the effect of TOD on imaging brightness we calculated the expected three-photon signal photons per laser pulse at a range of TOD values and normalized this value to the expected three-photon signal photons per laser pulse for a laser pulse with no TOD [see Sec. 2 and Eq. (2)]. This fractional photons per pulse shows a decrease to less than half the original brightness using our measured value of $22,750\;{\mathrm{fs}}^{3}$, and progressively less brightness as TOD is further increased [Fig. 4]. Using a higher average laser power to recover this loss in brightness comes with additional detriments of potential heat damage, which will limit the duration of imaging and/or the penetration depth. Three-photon microscopy users attempting to maximize fluorescence brightness and minimize collateral heating^20^^–^^22^ will benefit most from systems that have laser pulses with minimal third (and higher)-order phase distortions.

The detrimental effects of these distortions can be partially mitigated by utilizing laser sources without an excessive bandwidth (i.e., only enough bandwidth to reach the desired FTL). Although somewhat counter-intuitive, at large values of TOD, some brightness may be recovered by utilizing a slightly narrower bandwidth. However, choosing a narrower bandwidth comes with the cost of increasing the FTL. To quantify the effect of TOD on imaging brightness at a range of laser bandwidths, we calculated the expected three-photon signal photons per pulse over a range of TOD values and laser bandwidths using Eq. (2). We normalized these values to the expected three-photon signal photons per pulse of laser pulses with the zero TOD and the broadest bandwidth investigated [Fig. S3 in Supplementary Material]. It remains to be seen whether increasing bandwidth beyond this value will be beneficial due to the presence of non-optimal wavelength components and other practical constraints.^17^ These results suggest that if a three-photon laser source has a high amount of TOD, imaging quality could be improved most by utilizing a larger bandwidth, and reducing TOD with additional optics in the light path that can compensate specific phase profiles, e.g., with chirped mirrors.

Assuming phase distortions such as TOD are minimized such that the most efficient fluorescence excitation can be achieved, end-users employing three-photon imaging in their laboratories must still contend with pulse-to-pulse intensity fluctuations that may exist in the excitation laser pulses. Therefore, in the following section we propose a new technique for evaluating the energy and intensity stability of laser pulses over timescales relevant for three-photon imaging.

### 3.4 Diagnosing Pulse-to-Pulse Intensity Fluctuations and Visualizing their Effects in Imaging

#### 3.4.1 Imaging at the microscope

**Fig. 5:**
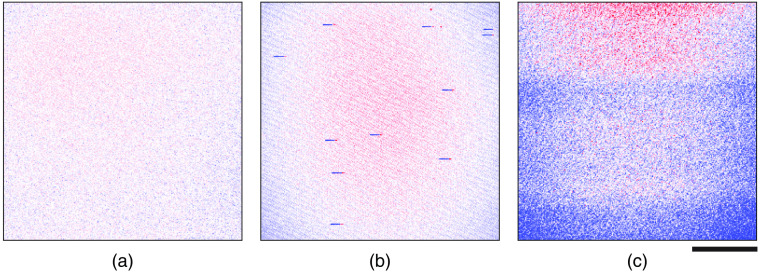
Three-photon microscopy images of a uniform fluorescent slide under normal conditions and in the presence of brightness artefacts. (a) Uniformly illuminated microscope image when the laser excitation source was optimized. In this and subsequent panels, minimum and maximum values of fluorescence in the image is scaled to ±95% of the mean such that red represents signals that are higher than the mean, white are at the mean, and blue are below the mean. (b) Image with intermittent microsecond-scale streaks of reduced brightness (dark blue pixels) followed by a very short rebound (red pixels). (c) Image with slower millisecond-scale temporal modulation of brightness. All images are 256×256  pixels, where each pixel represents the combined fluorescence signal from three laser pulses, i.e., an integration time or pixel dwell time of 3  μs from a laser with a repetition rate of 1 MHz. Scale bar 100  μm.

Several types of fluorescence artefacts on timescales relevant for imaging may be present in laser systems used for three-photon microscopy. Ideally, three-photon microscopy images of a uniformly fluorescent slide should present a field of view with relatively uniform brightness [Fig. 5(a)]. However, when artefacts on the order of ∼10 to 100  μs are present, they can manifest as short dark streaks in the microscope images [Fig. 5(b)]. Alternatively, when artefacts on the order of ∼10 to 100 ms are present in the microscope images, they can manifest as horizontal bright and dark banding [Fig. 5(c)]. The laser source used to generate each of the images in Fig. 5 was within specification with respect to RMS% average power stability (RMS% <2%). However, RMS% power stability measurements use thermopile sensors, which typically have a time resolution of ∼1  s. Thus, these measurements give no information on variations in pulse-to-pulse energy or intensity over the time scale of microseconds to milliseconds, which are the critical timescales for three-photon imaging.

#### 3.4.2 Quantitative strategy to monitor laser pulse-to-pulse stability

To test the pulse-to-pulse intensity stability of the laser, we developed a technique called deep-memory diode imaging (DMDI) (see Sec. 2). In DMDI, pulsed laser light is projected onto a photodiode and the photodiode signal is recorded by a high sampling rate oscilloscope with a large buffer to capture the pulse signal peaks at a high temporal resolution. To match the order of photo-absorption process in three-photon imaging, a tight-focusing element (e.g., a microscope objective) can be used to focus the laser onto a photodiode with an appropriately chosen band-gap that disallows one- and two-photon excitation [e.g., GaP; see Figs. S4(a) and S4(b) in Supplementary Material]. Like three-photon excited fluorescence, the photodiode signal recorded in this way is sensitive to peak pulse intensity and not just to total pulse energy. Alternatively, to simply capture the stability of pulse energy, a photodiode sensitive to one-photon excitation (e.g., InGaAs) may be used, which obviates the need for any focusing elements [Fig. S4(c) in Supplementary Material]. To localize the source of any laser instability, the one- or three-photon photodiode being used in DMDI can be placed at different locations in the optical path, such as under the microscope objective [Fig. S4(a) in Supplementary Material] or at the laser head [Figs. S4(b) and S4(c) in Supplementary Material]. Due to the use of high sampling rates and large buffer memory, DMDI can record relatively long and uninterrupted segments of pulse trains without aliasing of the signal [see Sec. 2 and Fig. S5 in Supplementary Material]. Therefore, DMDI offers a versatile and robust method to measure the laser pulse stability relevant for three-photon imaging.

#### 3.4.3 Linking photodiode measurements from the laser to microscopy images

**Fig. 6:**
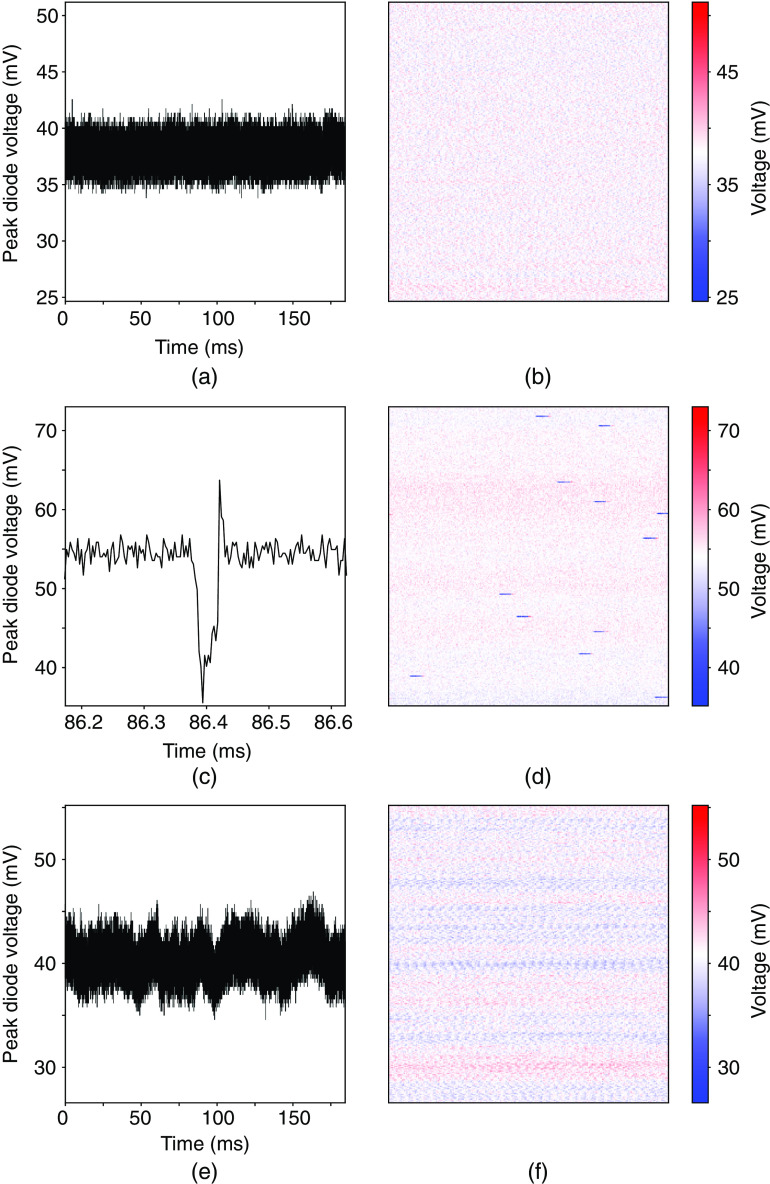
Direct measures of laser pulse intensity fluctuations using a photodiode. (a) and (b) Normal condition with stable laser pulses. (c) and (d) Microsecond-scale fluctuations of laser pulses with clear streaks of reduced brightness. (e) and (f) Millisecond scale wave-like fluctuations. For each of the three conditions, to mimic a pixel integration time of 3  μs that was used during imaging (as shown in Fig. 5), here the photodiode data are arranged as peak voltage signals, which were summed from every three successive pulses (left column). Then the same voltage signals are shown as 256×256  pixel images to visualize laser pulse intensity fluctuations in a manner akin to a microscope image (right column). The time axis in (c) and (e) are adjusted to reveal the relevant fluctuation patterns. The y axis and color bar in each case are scaled to 35% of the mean voltage.

We first used DMDI to monitor the pulse-to-pulse intensity stability of the laser by placing the three-photon photodiode (GaP) under the objective lens in our microscope [Fig. S4(a) in Supplementary Material]. Since the microscope images in Fig. 5 use a 3-μs integration time (and therefore each pixel represents the fluorescence signal of three laser pulses from a laser operating at 1 MHz repetition rate), the sum of three successive photodiode signal peaks was taken as a proxy for the brightness of one imaging pixel. This summed peak voltage signal was measured under conditions of artefact-free imaging and during imaging that had short and long-timescale artefacts. Plotting the time courses of summed peak voltage signals under these three conditions revealed an underlying fluctuation in pulse intensities or lack thereof [Figs. 6(a), 6(c), and 6(e)]. We then rearranged this linear array of summed peak voltages into a 256×256 matrix to resemble a three-photon image [Figs. 6(b), 6(d), and 6(f)]. Whether the image is generated in the traditional manner with the aid of a microscope or by DMDI, the same phenomenological fluctuations are clearly identified. This is readily demonstrated in comparing Fig. 5(b) with Fig. 6(d), and Fig. 5(c) with Fig. 6(f), which were obtained when using laser sources that had 10 to 100  μs fluctuations, and 10 to 100 ms fluctuations, respectively.

#### 3.4.4 DMDI in laser diagnosis and repair

Beyond attributing the imaging artefacts to pulse intensity fluctuations, DMDI aided our laser manufacturer in improving pulse-to-pulse stability. To this end, we performed DMDI at the laser head using a one-photon photodiode, revealing that the 10- to 100-μs intensity fluctuations stemmed from fluctuations in pulse energy originating from the laser source itself [see Fig. S6 in Supplementary Material]. The slower 10- to 100-ms intensity fluctuations may originate from several sources in addition to the laser, e.g., we found that air currents introduced in the beam path through small gaps (<1  mm) in beam-shielding tubes can contribute to these fluctuations.

#### 3.4.5 More sensitive statistic to describe laser pulse stability

Conventional statistics (e.g., RMS%, see Sec. 2) on data collected either with thermopile sensors or photodiodes used in DMDI may fail to capture potential pulse-to-pulse instabilities. For example, the RMS% of the signal peaks of the pulse trains in all three conditions shown in Fig. 6 was 3% to 6%. We found that another simple statistic is more sensitive to large departures from the mean, i.e., $\Delta \mu =100\times (S_{\max}-S_{\min})/\overset{¯}{S}$, where $S_{\max}$ is the largest signal peak, $S_{\min}$ is the smallest signal peak, and $\overset{¯}{S}$ is the average signal peak over the entire time course of the measurement. When calculated on the photodiode pulse train data over three recording periods, this “min-max” statistic (Δμ) was 42.5±1.4% (for the stable condition) and 76.9±2.4% (for the 10 to 100  μs “streak” artefacts). Statistics presented here are mean ± standard deviation. While RMS% measurements can capture large-scale oscillations in the amplitude variability of pulse trains, the min-max statistic better captures the momentary 10- to 100-μs deviations from the mean. In addition to providing an intuitive snapshot of laser performance communicated through the deep-memory diode image, computing the min-max statistic for data collected by DMDI provides a direct quantitative measurement of pulse-to-pulse stability over conventional methods such as RMS%.

## 4 Future Outlook

Although average power and pulse duration are measured to evaluate pulsed-laser performance as an industry standard, the equipment often used in these measurements (such as thermopile sensors and autocorrelators) do not capture the aspects of performance that are critical to three-photon microscopy. Due to few pulses being used for each imaging pixel and the cubic intensity dependence on fluorescence brightness per unit time, pulse-to-pulse intensity stability should be demonstrated in laser sources intended for three-photon microscopy (ideally using a technique such as DMDI). Additionally, laser pulses should be shown to have minimal higher-order phase using a spectral phase-resolved measurement (e.g., FROG) so that the laser pulses can be compressed and efficiently used in imaging. If pulse-to-pulse intensity stability is not demonstrated, three-photon imaging artefacts may be present. Finally, in laser sources that have large amounts of higher-order spectral phase (e.g., TOD), laser pulses with more energy will be needed to achieve the same peak intensity, which will ultimately limit the depth and the duration of three-photon imaging in biological samples due to average power limits.

## References

[1] Svoboda K. et al., “*In vivo* dendritic calcium dynamics in neocortical pyramidal neurons,” Nature 385, 161–165 (1997). 10.1038/385161a0 8990119

[r2] Ohki K. et al., “Functional imaging with cellular resolution reveals precise micro-architecture in visual cortex,” Nature 433, 597–603 (2005). 10.1038/nature03274 15660108

[3] Lu R. et al., “Rapid mesoscale volumetric imaging of neural activity with synaptic resolution,” Nat. Methods 17, 291–294 (2020).1548-7091 10.1038/s41592-020-0760-9 32123393 PMC7192636

[4] Busche M. A. et al., “Clusters of hyperactive neurons near amyloid plaques in a mouse model of Alzheimer’s disease,” Science 321, 1686–1689 (2008). SCIEAS 0036-8075 10.1126/science.1162844 18802001

[5] Ueki H. et al., “*In vivo* imaging of the pathophysiological changes and neutrophil dynamics in influenza virus-infected mouse lungs,” Proc. Natl. Acad. Sci. USA 115, E6622–E6629 (2018). 10.1073/pnas.1806265115 29941581 PMC6048509

[6] Ueki H. et al., “Multicolor two-photon imaging of *in vivo* cellular pathophysiology upon influenza virus infection using the two-photon IMPRESS,” Nat. Protoc. 15, 1041–1065 (2020).1754-2189 10.1038/s41596-019-0275-y 31996843 PMC7086515

[7] Hackl M. J. et al., “Tracking the fate of glomerular epithelial cells *in vivo* using serial multiphoton imaging in new mouse models with fluorescent lineage tags,” Nat. Med. 19, 1661–1666 (2013).1078-8956 10.1038/nm.3405 24270544 PMC3884556

[8] Horton N. G. et al., “*In vivo* three-photon microscopy of subcortical structures within an intact mouse brain,” Nat. Photon. 7, 205–209 (2013). NPAHBY 1749-4885 10.1038/nphoton.2012.336 PMC3864872 24353743

[r9] Ouzounov D. G. et al., “*In vivo* three-photon imaging of activity of GCaMP6-labeled neurons deep in intact mouse brain,” Nat. Methods 14, 388–390 (2017).1548-7091 10.1038/nmeth.4183 28218900 PMC6441362

[r10] Wang T. et al., “Three-photon imaging of mouse brain structure and function through the intact skull,” Nat. Methods 15, 789–792 (2018).1548-7091 10.1038/s41592-018-0115-y 30202059 PMC6188644

[r11] Takasaki K. Abbasi-Asl R. Waters J., “Superficial bound of the depth limit of two-photon imaging in mouse brain,” eNeuro 7, ENEURO.0255-0219.2019 (2020). 10.1523/ENEURO.0255-19.2019 PMC6984806 31907211

[12] Liu C. J. et al., “Three-photon imaging of synthetic dyes in deep layers of the neocortex,” Sci. Rep. 10, 16351 (2020). SRCEC3 2045-2322 10.1038/s41598-020-73438-w 33004996 PMC7529898

[13] Xu C. et al., “Multiphoton fluorescence excitation: new spectral windows for biological nonlinear microscopy,” Proc. Natl. Acad. Sci. USA 93, 10763–10768 (1996). 10.1073/pnas.93.20.10763 8855254 PMC38229

[14] Farinella D. M. et al., “Demonstration of thin film compression for short-pulse X-ray generation,” Int. J. Mod. Phys. A 34, 1943015 (2020). IMPAEF 0217-751X 10.1142/S0217751X19430152

[15] Jafari R. Jones T. Trebino R., “100% reliable algorithm for second-harmonic-generation frequency-resolved optical gating,” Opt. Express 27, 2112–2124 (2019). OPEXFF 1094-4087 10.1364/OE.27.002112 30732254

[16] Hult J., “A fourth-order Runge–Kutta in the interaction picture method for simulating supercontinuum generation in optical fibers,” J. Lightwave Technol. 25, 3770–3775 (2007). JLTEDG 0733-8724 10.1109/JLT.2007.909373

[17] Wang T. Xu C., “Three-photon neuronal imaging in deep mouse brain,” Optica 7, 947–960 (2020). 10.1364/OPTICA.395825

[18] DeLong K. W. et al., “Frequency-resolved optical gating with the use of second-harmonic generation,” J. Opt. Soc. Am. B 11, 2206–2215 (1994). JOBPDE 0740-3224 10.1364/JOSAB.11.002206

[19] Klioutchnikov A. et al., “Three-photon head-mounted microscope for imaging deep cortical layers in freely moving rats,” Nat. Methods 17, 509–513 (2020).1548-7091 10.1038/s41592-020-0817-9 32371979

[20] Yildirim M. et al., “Functional imaging of visual cortical layers and subplate in awake mice with optimized three-photon microscopy,” Nat. Commun. 10, 177 (2019). NCAOBW 2041-1723 10.1038/s41467-018-08179-6 30635577 PMC6329792

[r21] Wang T. et al., “Quantitative analysis of 1300-nm three-photon calcium imaging in the mouse brain,” eLife 9, e53205 (2020). 10.7554/eLife.53205 31999253 PMC7028383

[22] Li B. et al., “An adaptive excitation source for high-speed multiphoton microscopy,” Nat. Methods 17, 163–166 (2020).1548-7091 10.1038/s41592-019-0663-9 31792434 PMC7004891

