# Supplementary material for: Improving laser standards for three-photon microscopy
Source: Neurophotonics. 2021 Mar 6;8(1):015009. doi: 10.1117/1.NPh.8.1.015009 (PMC7937945; doi:10.1117/1.NPh.8.1.015009)
Supplement: Supplementary file 1 [file NPh_008_015009_SD001.pdf]

SUPPLEMENTARY INFORMATION INTENDED FOR PUBLICATION

# Table of Contents

*Supplementary Figures*........................................................................................................2

*List of Abbreviations*........................................................................................................8

# Supplementary Figures

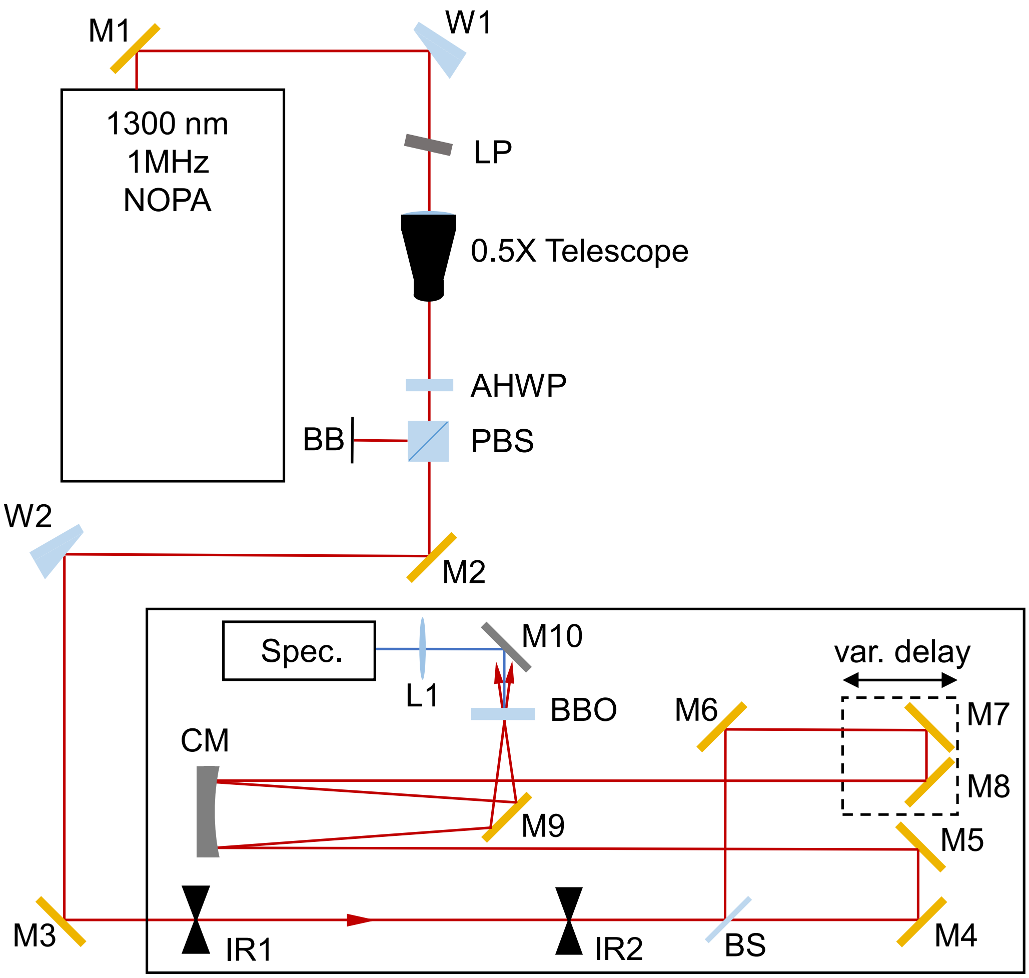


**Fig. S1.** Schematic of light path and scanning SHG FROG setup. W1 and W2: uncoated fused silica wedges, LP: long-pass filter, AHWP: achromatic half-wave plate, PBS: polarizing beam splitter, BB: beam block, 0.5X Telescope: C-coated 0.5X down-collimating telescope, IR1 and IR2: irises, BS: pellicle beam splitter, CM: curved mirror, BBO: SHG crystal (β-Barium Borate), L1: BK7 lens, Spec.: Spectrometer, var. delay: retro-reflecting mirror pair mounted on a translation stage. Mirrors drawn as gold symbols are gold-coated and those shown in grey are silver-coated.

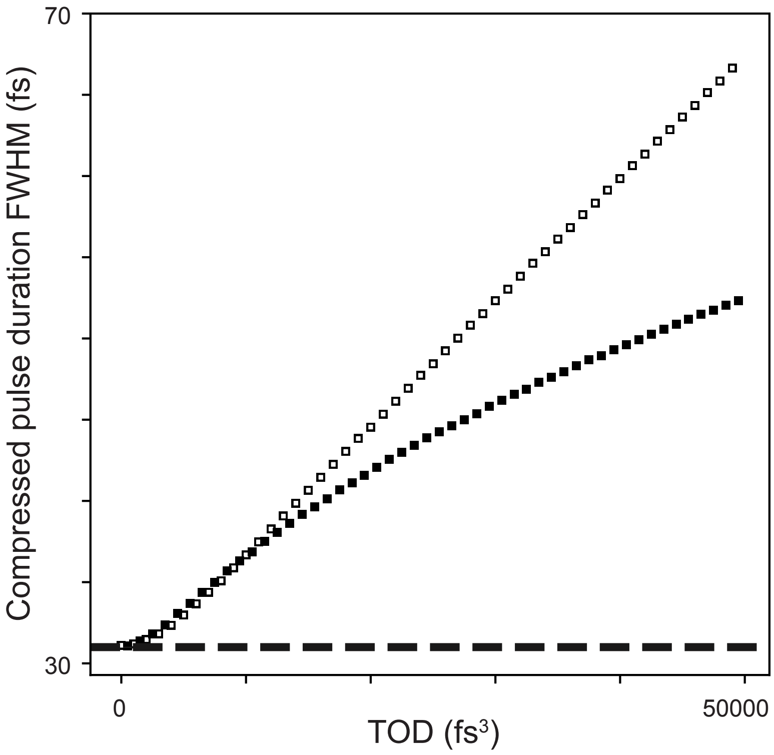


**Fig. S2**. Limitations of using autocorrelations for measuring laser pulses that have TOD. Filled black squares represent the FWHM of the temporal intensity envelope of our model laser pulses (31 fs FTL) with a range of TOD values and no other phase distortions such as GDD. Unfilled black squares represent the pulse duration that would be measured for these same pulses by autocorrelations, if the underlying pulse shape was assumed to be Gaussian. The horizontal dashed line represents a perfectly compensated laser pulse with no phase distortions.

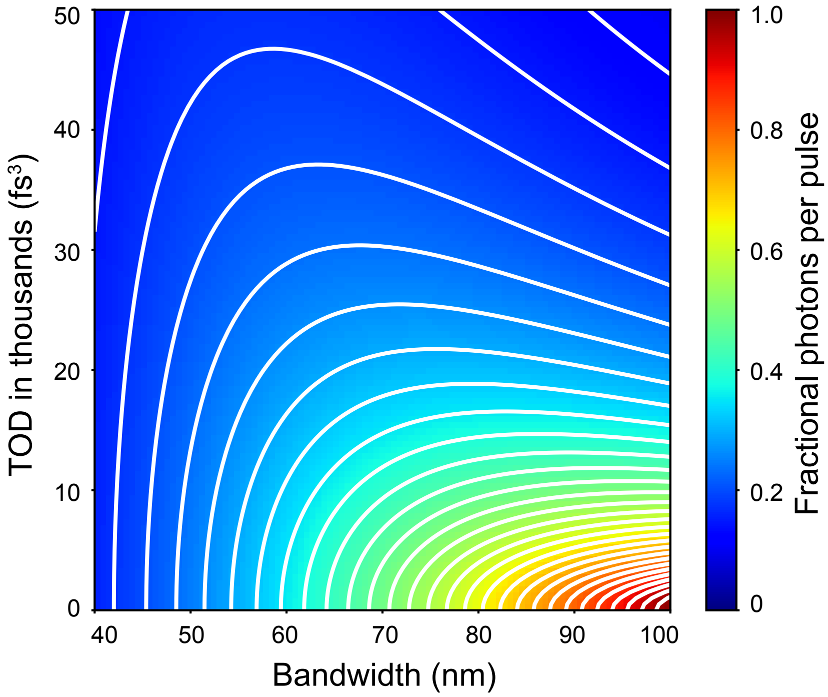


**Fig. S3.** The effect of TOD on three-photon imaging brightness evaluated at a range of laser bandwidths for GDD compensated pulses. Gaussian laser pulses at a range of laser bandwidths were given a range of TOD values with no other phase distortions (i.e., GDD = 0 $fs^2$). The resulting temporal profiles in each case were used to calculate the expected signal photons per pulse, and normalized to the case with the most bandwidth and the least TOD to give fractional photons per pulse. Contour lines represent lines of equal brightness.

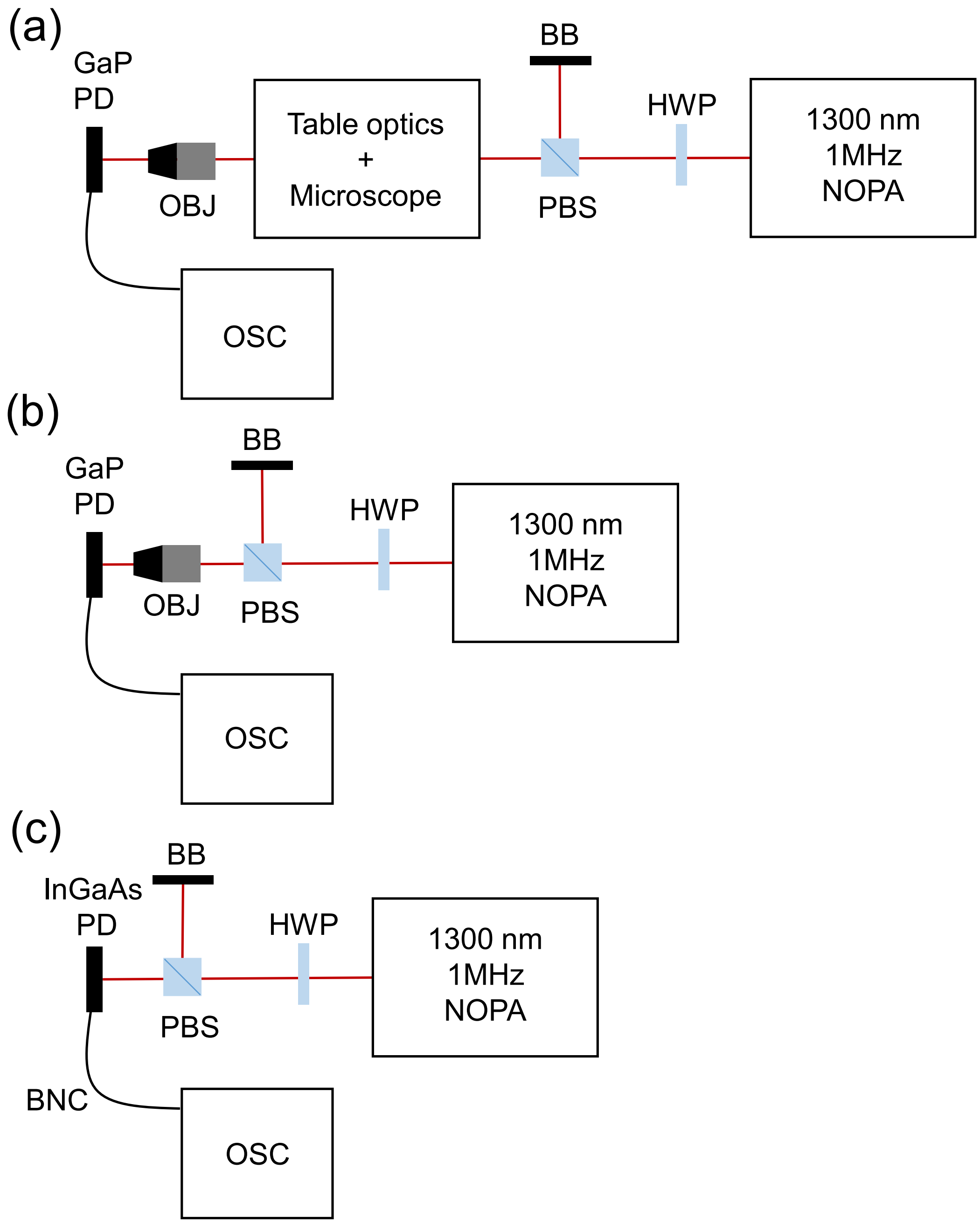


**Fig. S4**. Setup for deep memory diode imaging (DMDI) from our laser source. (a) Three-photon photodiode recording using table and microscope optics. (b) Three-photon photodiode recording directly from the laser source with the exception of minimal components which were necessary to control laser power. (c) One-photon photodiode recording setup from the laser source, which by definition, does not require an objective lens to focus the light on the sensor. PD: photodiode, BB: beam block, PBS: polarizing beam splitter, HWP: half wave plate, OSC: large memory buffer oscilloscope, NOPA: non-collinear optical parametric amplifier, OBJ: objective lens.

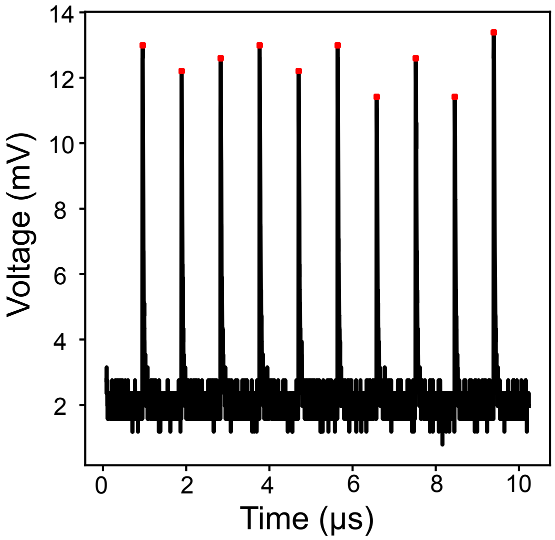


**Fig. S5.** An example time course of a raw voltage trace of three-photon photodiode signals using our high bandwidth digital oscilloscope [see Methods and Supplementary Fig. S4 (a)]. Each red dot represents the peak voltage detected using the “find_peaks” function in the signal processing toolbox of the open source Python library SciPy.

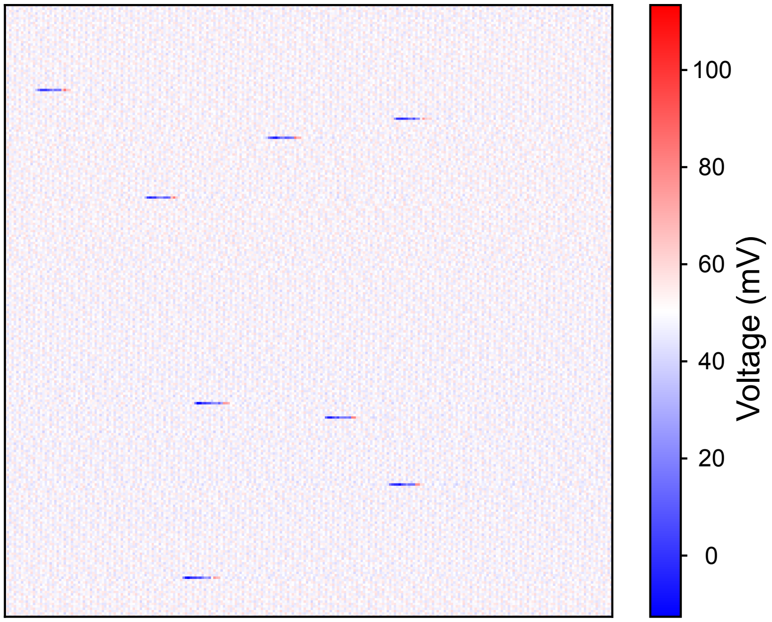


**Fig. S6**. Sample DMDI measurement of laser instability using a one-photon absorption diode. Each pixel represents the sum of three voltage peaks. The 2D image representation is a 256 × 256 array of pixels to mimic a microscope image. Minimum and maximum values of the lookup table were scaled to ±125% of the mean such that red represents voltage peaks that are higher than the mean, white represents peaks that are at the mean, and blue represents peaks that are below the mean.

# List of Abbreviations

GDD: Group delay dispersion
TOD: Third order dispersion
DMDI: Deep-memory diode imaging
FTL: Fourier transform limit
SHG FROG: Second harmonic generation frequency resolved optical gating
FWHM: Full width at half maximum
